# Knowledge, Attitude and Practice of Hospital Pharmacists in Central China Towards Adverse Drug Reaction Reporting: A Multicenter Cross‐Sectional Study

**DOI:** 10.3389/fphar.2022.823944

**Published:** 2022-03-22

**Authors:** Wen Hu, Yun Tao, Yun Lu, Suyu Gao, Xuanxuan Wang, Wenjing Li, Qiaoli Jiang, Likai Lin, Feng Sun, Hong Cheng

**Affiliations:** ^1^ Department of Pharmacy, Zhongnan Hospital of Wuhan University, Wuhan, China; ^2^ Hospital Management Institute of Wuhan University, Zhongnan Hospital of Wuhan University, Wuhan, China; ^3^ Department of Epidemiology and Biostatistics, School of Public Health, Peking University, Beijing, China

**Keywords:** adverse drug reaction reporting, hospital pharmacists, knowledge, attitude, practice, China

## Abstract

**Background:** Healthcare professionals’ knowledge and attitudes towards adverse drug reactions (ADRs) and ADR reporting play a significant role in pharmacovigilance. This study aims to investigate the gap between knowledge and practice in ADR reporting among hospital pharmacists.

**Methods:** This study is a multi-center, cross-sectional study based on a questionnaire survey. A semi-structured questionnaire was developed including knowledge, attitudes, and practices (KAP) towards ADR reporting. From October to November 2021, questionnaires were filled out on the internet by hospital pharmacists from a central province of China. The data analysis used a one-way ANOVA to analyze the differences between the pharmacist’s characteristics and knowledge and attitude, respectively. The ordinal logistic regression method was used to analyze the predictors of practice.

**Results:** A total of 1,026 valid questionnaires from 512 medical institutions were collected. It was found that 88.8% of participants have a clear understanding of the ADR definition, while 59.6% of them have misunderstandings about the reporting time of new and serious adverse reactions. Most pharmacists showed positive attitudes towards ADR reporting. Higher education background, higher professional title, attending training, and clinical pharmacist resulted in higher knowledge scores. Higher education background, shorter working years, attending training, and from non-tertiary hospital related to higher attitude scores. In terms of practice, age, hospital type, working years, training, and pharmacist type all have significant associations with practice scores. Pharmacists’ knowledge score and attitude score were significant predictors of practice score with OR being 1.19 (95% CI: 1.06, 1.33) and 1.04 (95% CI: 1.005, 1.07).

**Conclusion:** Although most hospital pharmacists showed positive attitudes towards ADR reporting, their knowledge and practice were still insufficient. Hospital pharmacists’ knowledge and attitude are associated with their practice towards ADR reporting. The training had a significant impact on the pharmacist’s knowledge, attitude, and practice.

## Introduction

The World Health Organization (WHO) defines adverse drug reactions (ADRs) as harmful and unrelated to the purpose of medication when normal doses of drugs are used to prevent, diagnose, treat diseases or regulate physiological functions (Edwards and Aronson, 2000). ADR has a significant impact on the health of patients, and is a major problem that has led to an increase in global morbidity and mortality. It is estimated that about 5% of hospitalized patients are caused by ADR, and another 5% of hospitalized patients will experience ADR during hospitalization. In the European Union, ADR causes 197,000 deaths every year (Bouvy et al., 2015). In the United States, the total cost of hospitalization after adverse drug events in the intensive care unit (ICU) and non-ICU ward is estimated to be 19,685 dollars and 13,994 dollars, respectively (Cullen et al., 1997). Therefore, monitoring adverse drug reactions are critical for global health care.

In all countries, national pharmacovigilance systems rely primarily on spontaneous reporting, in which suspected adverse drug reactions are reported to a national coordinating center by health professionals, pharmaceut post-marketing ical producers, or individuals (Pal et al., 2013). Spontaneous reports of ADRs have some advantages for identifying potential safety signals, but they have apparent drawbacks, such as substantial underreporting, poor report quality, difficulty quantifying risk, and an unknown number of people who have been exposed (Alharf et al., 2018; Hazell and Shakir, 2006).

China has a nationwide ADR reporting and monitoring system, composed of four levels, which includes the National Center for ADR monitoring, 34 provincial ADR monitoring centers, and hundreds of municipal and county-level institutions (Li et al., 2018; Zhao et al., 2018). In China, the number of spontaneous ADR reports was 1.7 million in 2020, which equates to 1215 reporting per million people. With a population of 1.4 billion people, China is attempting to expand the number of spontaneous ADR reports (Song et al., 2022).

The majority of ADR reports in China come from healthcare professionals (85.4%) (NMPA, 2020). According to a survey in three provinces in China, pharmacists reported the largest proportion of ADRs (43.51%) among all sources during 2015–2017, however, the quality of ADR reporting by pharmacists was not promising, with only 11.5% of reports being of high quality (Chen et al., 2019). Another investigation showed that hospital pharmacists in a northern province of China have good knowledge and attitudes but poor practice towards ADR reporting (Su et al., 2010). To better understand the challenges pharmacists face in reporting ADRs and to offer suggestions for improving the rate and quality of ADR reporting, we conducted this survey on pharmacists’ knowledge, perceptions and practice of ADR reporting.

## Study Design and Methods

### Participants and Setting

This is a multi center, cross-sectional study based on a questionnaire survey. The questionnaire was distributed to hospital pharmacists in QQ groups and WeChat groups in Hubei Province through the questionnaire collection software (Questionnaire Star), and a statement on the research project and consent form was distributed at the same time. From October to November 2020, a total of 1,128 people participated in the survey. The data was excluded based on the following criteria: I. Pharmacists who are not hospital pharmacists; II. Invalid. The questionnaire takes less than 1 min or more than 1 h to complete. In the end, a total of 1026 valid questionnaires were obtained, with an 89.7% effective rate. Participants were from 522 hospitals in Hubei Province. Participants’ responses are completely anonymous and voluntary.

### Questionnaire Design

The self-administered questionnaire was composed of 25 mandatory single-choice items and one multiple-choice item, and it was developed based on scientific literature (Chatterjee et al., 2006; Al Dweik et al., 2017; Seid et al., 2018) and the practice experience of the authors. Two experts in pharmacovigilance reviewed the questionnaire draft and assessed its content validity. The questionnaire consists of five main parts: (i) Pharmacist characteristics: education, profession rank, and length of work experience, etc.; (ii) Knowledge part: definition of ADR, ADR reporting time, etc., we set multiple-choice questions, each question has a correct answer, and the correct answer receives 1 point, while the incorrect answer receives 0 point; (iii) Attitudes part: concerning and willingness about ADR reporting, this part was provided on a 5-level Likert scale (1 = “strongly disagree,” 2 = “disagree,” 3 = “neutral,” 4 = “agree,” and 5 = “strongly agree”) to indicate that they disagreed or agreed.; (iv) Practice part: covers two items based on the surveyors’ ADR reporting practice experience, we set up a yes or no question option, “yes” gets 2 points, “no” gets 1 point; (v) Part five: investigated the influencing factors for ADR reporting and was provided in selective form. Detailed explanations and correct answers were in Supplementary Material S1.

### Data Processing

For describing demographic variables, descriptive statistics are used, using percentages or frequencies to demonstrate categorical variables. One-way ANOVA was used to explore the relations between pharmacists’ characteristics and knowledge and attitude scores, respectively, and ordinal logistic regression was used to analyze the correlation between knowledge, attitude and practice. The characteristic factors with *p* < 0.05 in the single factor analysis results were taken as covariates in the ordinal logistic regression. SPSS 22.0 was used for statistical analysis. *p* < 0.05 was considered statistically significant.

## Results

### Characteristics of Pharmacist

A total of 1,128 questionnaires were collected, and 1,026 of them were valid and included in the analysis. The effective rate was 89.1%. There were 335 (32.7%) males and 691 females (67.3%); 437 pharmacists (42.6%) were under the age of 35; 512 pharmacists (49.9%) were from non-tertiary medical institution; 950 pharmacists (92.6%) have undergraduate degrees; and 569 pharmacists (55.5%) have intermediate professional titles. 837 pharmacists (81.6%) had more than 6 years of work experience; 778 pharmacists (75.8%) had participated in ADR training before; and there were 199 clinical pharmacists (19.4%). The characteristics of pharmacists are showed in Table 1.

**TABLE 1:** Characteristics of pharmacists.

Variables	N	Percentage (%)
Respondents	1026	—
Gender		
Male	335	32.7%
Female	691	67.3%
Age (years)		
≤35	437	42.6%
36–45	277	27.0%
>45	312	30.4%
Type of Medical institution		
Tertiary hospital	514	50.1%
Non-tertiary hospital	512	49.9%
Education		
High school and below	76	7.4%
College degree	795	77.5%
Master degree and above	155	15.1%
Professional rank		
Junior	332	23.4%
Intermediate	569	55.5%
Senior	125	12.2%
Years of working		
≤5	189	18.4%
6–20	440	42.9%
>20	397	38.7%
Training attending		
Yes	778	75.8%
No	248	24.2%
Types of Pharmacists		
Clinical pharmacist	199	19.4%
Dispensing pharmacist	827	80.6%

### Pharmacist’s Knowledge of ADR

**FIGURE 1:**
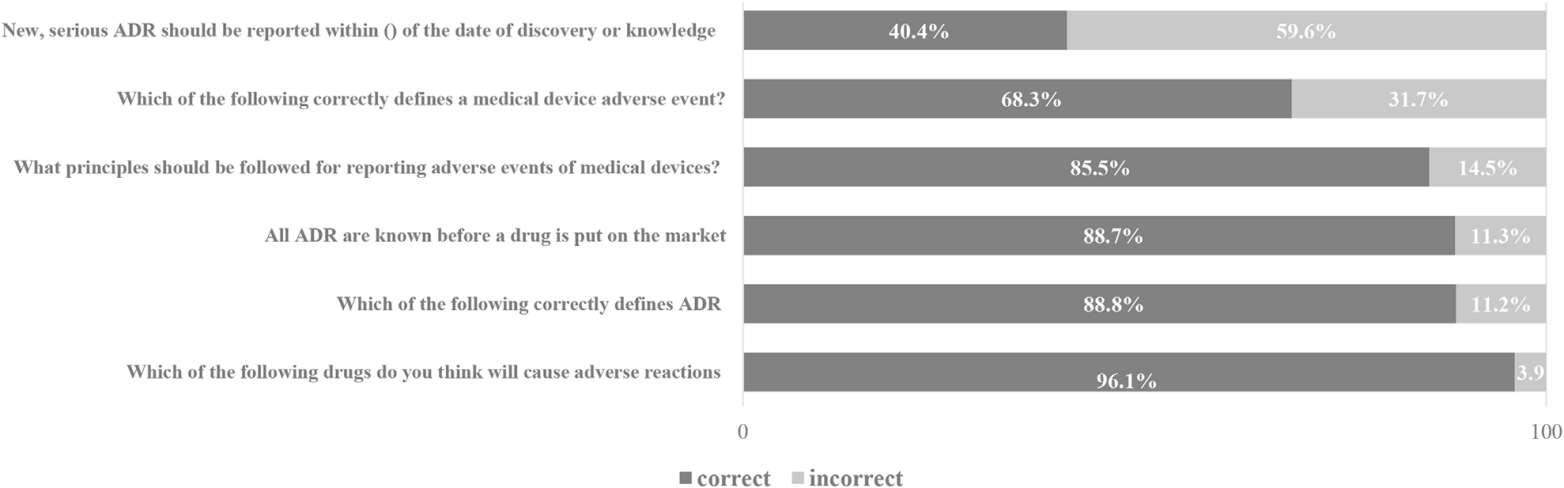
Knowledge of pharmacists toward ADR.

The results of pharmacists’ knowledge of ADR reporting showed that 88.8% of people had a clear understanding of the definition of adverse drug reactions, while 59.6% had misunderstandings about the reporting time of new and serious adverse reactions. At the same time, 31.7% of pharmacists have cognitive errors in the definition of medical device adverse events, and 14.5% have cognitive errors in the reporting principles of medical device adverse events. The pharmacist’s knowledge scores on ADR reporting are shown in Figure 1.

### Pharmacist’s Attitude Towards ADR

The results of the pharmacist’s attitude towards ADR reporting indicate that the majority of pharmacists have a positive attitude towards ADR reporting. 95.1% agreed that adverse drug reaction monitoring is beneficial to public health, 84.9% believed that adverse drug reaction reporting was part of their responsibilities, 91.2% disagreed that only serious adverse drug reactions were reported, and 94.5% were willing to participate in adverse drug reaction reporting training. Regarding the issue of whether the adverse drug reaction report will generate additional workload, 48.4% of people believe that ADR reporting will generate additional workload. The results are shown in Table 2.

**TABLE 2:** Attitudes of pharmacists towards ADR.

Items	Strongly agree (%)	Agree (%)	Neutral (%)	Disagree (%)	Strongly disagree (%)
You will pay attention to the possible ADR of patients	38.0	49.9	10.5	0.4	1.2
Do you agree that monitoring of ADR is beneficial to public health?	59.6	35.5	1.6	0.9	2.5
Do you agree that reporting an ADR report can also have an impact?	38.0	49.9	10.5	0.4	1.2
Do you consider reporting ADR as part of your responsibilities?	35.0	49.9	10.5	0.4	1.2
Do you think that only serious ADR should be reported?	0.9	3.6	4.4	62.4	28.8
Do you think that the ADR report will generate extra workload?	12.9	35.5	22.6	24.7	4.4
Are you willing to participate in the training of ADR reports?	38.5	56.0	4.8	0.2	0.5
Do you think that monitoring of adverse drug reactions should protect patient privacy?	38.4	56.0	4.8	0.3	0.5
Do you think that adverse drug reactions should be reported regularly?	38.5	56.0	4.8	0.2	0.5

### Pharmacist’s Practice of ADR Reporting

According to the results of ADR reporting, 70.9% of pharmacists had encountered adverse drug reactions, of which 67.3% had reported adverse drug reactions.

### Differences Between Pharmacists’ Characteristics and KAP Towards ADR

The study found that there was a significant difference between pharmacists’ characteristics and ADR knowledge scores. Among them, pharmacists’ education, professional title, whether they participate in training, and job types have significant differences in ADR cognitive scores (*p* < 0.05). Pharmacists with a high school degree or below, junior professional titles, pharmacists who have not participated in training, and dispensing pharmacists have relatively low knowledge scores. The knowledge scores of pharmacists in non-tertiary hospitals were lower than those in tertiary hospitals (*p* < 0.05). There is a significant difference between pharmacist characteristics and ADR attitude scores. Among them, male, younger than 35 years old, non-tertiary hospital, low educational background, less than 5 years of work experience, and pharmacists with no training have relatively lower scores (*p* < 0.05). The differences in knowledge and attitude of pharmacists are shown in Table 3.

**TABLE 3:** The relation between the pharmacist’s characteristics and KAP.

Variable	Knowledge score (0–6)			Attitude score (0–45)		
	Average	SD	*p*	Average	SD	*p*
Gender					
Male	4.74	1.11	0.29	36.83	4.37	**0.042**
Female	4.66	1.18		37.37	3.75	
Age (years)					
≤35	4.72	1.19	0.72	37.39	4.10	**0.008**
36–45	4.65	1.20		37.52	3.85	
>45	4.66	1.09		36.62	3.83	
Type of hospital					
Tertiary	4.74	1.14	0.123	37.47	4.04	**0.024**
Non-tertiary	4.63	1.18		36.91	3.89	
Education background					
High school and below	4.08	1.23	**<0.001**	35.83	3.64	**0.007**
College degree	4.66	1.18		37.27	4.00	
Master degree and above	5.10	0.85		37.45	3.89	
Professional rank					
Junior	4.51	1.29	**0.001**	37.21	4.03	0.983
Intermediate	4.73	1.11		37.17	3.92	
Senior	4.91	0.95		37.23	4.08	
Working years					
≤5	4.67	1.25	0.68	37.49	4.01	**0.033**
6–20	4.72	1.16		37.43	4.09	
>20	4.65	1.12		36.79	3.79	
Training attending					
Yes	4.73	1.18	**0.014**	37.48	4.04	**0.00**
No	4.52	1.08		36.29	3.60	
Type of pharmacist					
Clinical pharmacist	5.07	1.01	**<0.001**	37.35	4.06	0.54
Dispensing pharmacist	4.59	1.18		37.16	3.95	

The bold values are statistically significant data, mainly for ease of viewing.

### The Predictors of Pharmacists’ Practice Toward ADR Reporting

The knowledge and attitude scores were used as predictors of pharmacists’ practice toward ADR reporting to explore the relationship within KAP (Table 4). A significant association was observed for both knowledge [OR (95% CI): 1.19 (1.06, 1.33), *p* = 0.002] and attitude score [OR (95% CI): 1.04 (1.005, 1.07), *p* = 0.023] with the practice score of pharmacists. The covariates of the model are gender, age, hospital grade, educational background, title, working years, whether to participate in training, and job type. Pharmacist characteristics were also predictors of practice scores. The results of the multivariate model revealed that the risks of having a higher practice score were 0.60 (95% CI: 0.39, 0.94) times higher among pharmacists belonging to the age group 36–45 years, when compared with pharmacists older than 45 years. Pharmacists from non-tertiary hospitals had 1.64 (95% CI: 1.24, 2.17) times greater risk of having higher scores in practice compared with pharmacists from tertiary hospitals. The risks of having a higher practice score were 2.98 (95% CI: 1.59, 5.59) times higher among pharmacists belonging to the working year group ≤5 compared with ≥20 years. Pharmacists with training experience had 1.75 (95% CI: 1.30, 2.35) times higher practice scores compared with pharmacists with no-training experience. Dispensing pharmacists have 0.22 (95% CI: 0.14, 0.35) times higher practice scores than clinical pharmacists.

**TABLE 4:** Predictors of practice.

Variables	Practice score	
	OR (95% CI)	*p*-value
Knowledge score	1.19 (1.06, 1.33)	**0.002**
Attitude score	1.04 (1.005, 1.07)	**0.023**
Gender		
Male	0.95 (0.72, 1.25)	0.718
Female	Ref	
Age (years)		
≤35	0.58 (0.33, 1.03)	0.064
36–45	0.60 (0.39, 0.94)	**0.026**
>45	Ref	
Type of hospital		
Tertiary	Ref	**0.001**
Non-tertiary	1.64 (1.24, 2.17)	
Education background		
High school and below	0.87 (0.43, 1.75	0.69
College degree	1.21 (0.78, 1.89)	0.40
Master degree and above	Ref	
Professional title		
Junior	1.64 (0.93, 2.91)	0.09
Intermediate	1.34 (0.83, 2.16)	0.23
Senior	Ref	
Working years		
≤5	2.98 (1.59, 5.59)	**0.001**
6–20	1.34 (0.84, 2.16)	0.223
>20	Ref	
Training attending		
Yes	1.75 (1.30, 2.35)	**<0.001**
No	Ref	
Type of pharmacist		
Clinical pharmacist	Ref	**<0.001**
Dispensing pharmacist	0.22 (0.14, 0.35)	

The bold values are statistically significant data, mainly for ease of viewing.

### Factors Affecting Pharmacists’ Reporting of ADRs

**FIGURE 2:**
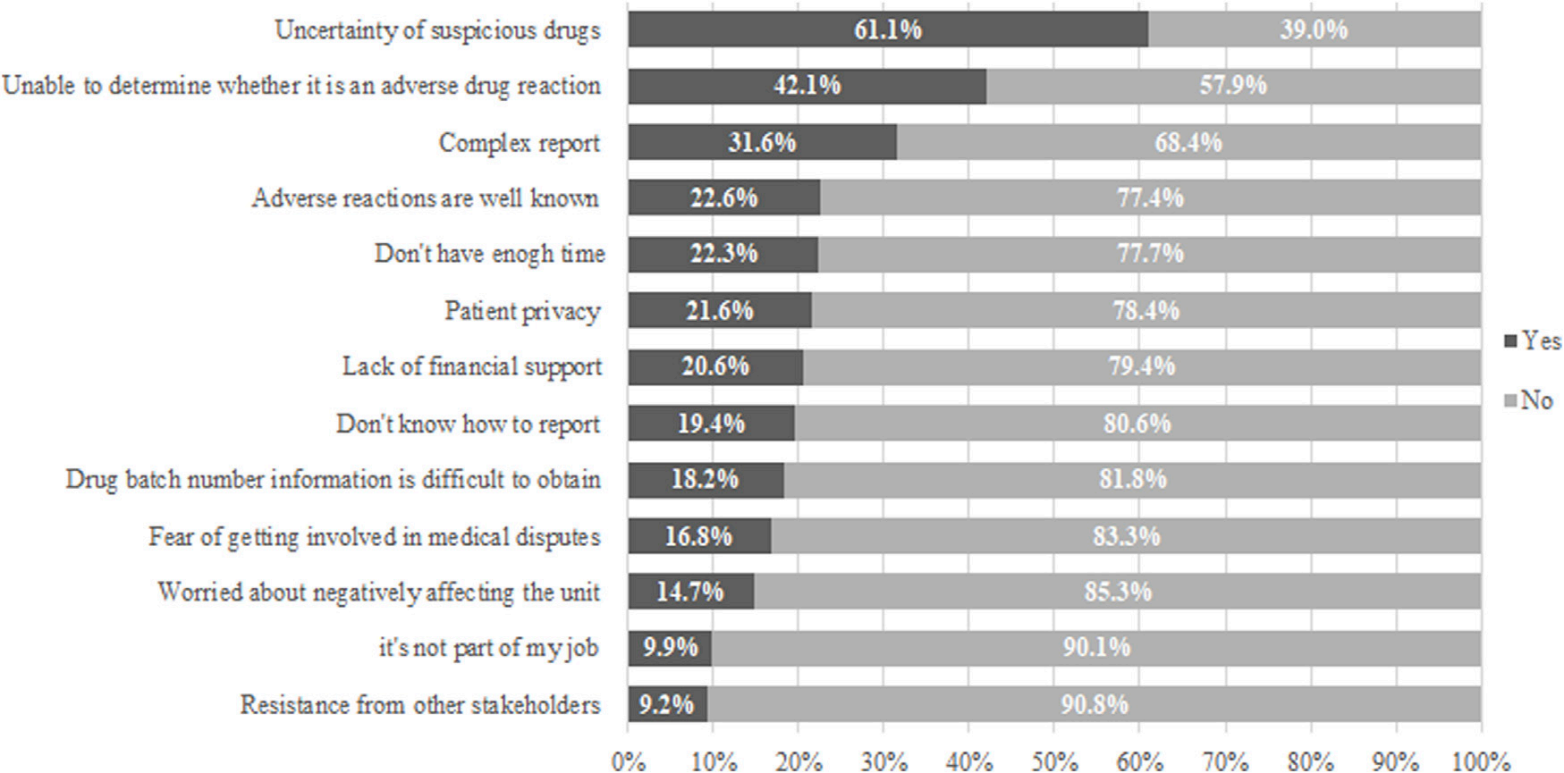
Factors influencing the reporting of ADR among pharmacists.

Our study also investigated the factors affecting pharmacists’ ADR reporting (Figure 2). The investigation’s findings indicated that the first three main factors affecting pharmacists’ ADR reporting were the uncertainty about the suspected drug, the inability to determine whether it was an adverse drug reaction, and the report’s complexity. At the same time, 24.7% of people don’t know how to report.

## Discussion

### The Importance of Hospital Pharmacists in the ADR Reporting Process

Drugs are the most common treatment for diseases, so it is necessary to pay attention to the rational use of drugs. If the safety of the drug is not considered properly, it may lead to consequences ranging from lifelong disability to death. Similarly, if a drug-related ADR is reported, the safety of the drug can be improved (Babar and Jamshed, 2008). This study aims to evaluate and compare the differences in knowledge, attitude, and practice of ADR reporting among pharmacists with different characteristics. Since many serious ADRs occur in hospitals or lead to hospitalization, pharmacists in medical institutions play an important role in ADR reporting (Lazarou et al., 1998). At the same time, most new drugs will first be used in hospitals. Therefore, the research on the knowledge, attitude, and practice of hospital pharmacists in the ADR reporting process is particularly important.

### Hospital Pharmacists’ Knowledge, Attitude and Practice Characteristics

In this study, we found that 11.2% of pharmacists are still unclear about the basic definition of ADR, which is similar to the results of previous research reports (Su et al., 2010). 59.6% of pharmacists have misunderstandings about the reporting time of new and serious adverse reactions. It reflects that pharmacist still lacks basic knowledge about ADR reports. Among them, pharmacists with low academic qualifications, low professional titles, and untrained pharmacists have relatively little basic knowledge related to ADR reporting. It reflects that pharmacist still lacks knowledge about ADR reporting. Among them, educational backgrounds, professional titles, and whether they have participated in the training are related to the basic knowledge related to ADR reporting. Highly educated and trained pharmacists are more familiar with the basic knowledge related to ADR reporting. In addition, the survey showed that 40% of the pharmacists had poor knowledge about medical device adverse events. This indicates that pharmacists need to be properly trained for ADR reporting so that the quality of ADR reporting can be improved.

Pharmacists’ attitude is considered the key to reporting ADR, so a positive attitude may encourage timely reporting of ADR. In the current study, pharmacists have a very positive attitude towards reporting ADR. Most pharmacists agree that ADR reporting is part of their responsibilities, which is consistent with the results of other similar studies (Hallit et al., 2019; Kopciuch et al., 2019; Al-Mutairi et al., 2021). Although most pharmacists have no significant differences in their attitudes towards ADR reporting, male pharmacists or under 35 years of age have slightly lower positive attitudes towards ADR reporting. It may be related to the new employee’s relatively short length of service. Since pharmacists who have worked for a long time have been exposed to more adverse events at work, they have a better understanding of the harm that adverse events can cause to patients. And get more training and assessment related to ADR reporting in the workplace. Therefore, they pay more attention to the harmfulness of ADRs and their attitude towards ADR reporting are more positive. The positive attitude among pharmacists who have participated in ADR training is higher. Most of the participants expressed interest in ADR report training, indicating that they believe it is important to learn more about ADR reporting. In fact, it is also possible that the participants are unwilling to present their problems due to the deviation of social expectations. Because of the importance of pharmacists’ knowledge and positive attitude towards ADR reporting, ADR administrative centers at all levels needs to strengthen training and education in the field of ADR.

In the current study, there is a significant difference between pharmacist characteristics and ADR practice scores. The difference of education level mainly affects the score of pharmacists’ basic knowledge of adverse reactions. Among them, only pharmacists with education below senior high school have a downward trend in the score of reporting attitude towards adverse drug reactions. It indicates that the educational level of some pharmacists needs to be improved. Lower practice scores are found amount pharmacists who are over 45 years old, have less than 5 years of experience, or have not participated in ADR training. Explain that work experience and ADR training have a greater impact on the practice of ADR reporting. Interestingly, pharmacists’ practice scores in tertiary hospitals are low, which may be related to the work nature of pharmacists in tertiary hospitals. Most pharmacists in tertiary hospitals are not only engaged in pharmacotherapeutic work, but also conduct research and teach, which diverts their attention away from observing ADRs in patients. Clinical pharmacists in Chinese hospitals are typically the ones who handle ADR reporting and are better prepared than dispensing pharmacists to detect and report ADRs (Chen et al., 2015). That could explain why dispensing pharmacists have a lower ADR reporting practice score than clinical pharmacists.

### The Relationship Between Pharmacist Characteristics and KAP

This study shows that both knowledge and attitude have a positive effect on the practice of pharmacists, and future improvement strategies can be carried out from the aspect of improving pharmacists’ knowledge and attitude towards ADR monitoring. As a strategy to improve the ADR reporting, it should be aimed at healthcare professionals’ level and the pharmacist level. In addition to encouraging pharmacists to report ADR, continuous professional development plans should be used to make up for their lack of knowledge and skills in discovering and reporting ADR. In this study, the main factors affecting pharmacists’ reports of adverse reactions are the uncertainty of suspected drugs, the inability to judge whether they belong to adverse drug reactions and the complexity of the report. There is evidence that providing continuing education to health professionals can help change their behavior and attitudes towards ADR reports (Gonzalez-Gonzalez et al., 2013; Pagotto et al., 2013). The purpose of such education should not only be limited to improving pharmacists’ knowledge of ADR but also be aimed at changing their attitudes and views on ADR report. The results of this study also show that pharmacists who have participated in ADR training have higher knowledge, attitude, and practice scores. As experts, pharmacists play an important role in ensuring drug safety by detecting and reporting ADRs (Hadi et al., 2017). In the past few decades, the role of pharmacists has changed worldwide, from dispensers to guardians of drug safety (Su et al., 2010; Oreagba et al., 2011; Hadi et al., 2017). Research evidence shows that hospital pharmacists can not only detect and report ADRs but also help prevent ADR-related occurrences. In addition, pharmacists who have a clinical background and work closely with prescribers and patients can better understand suspicious ADRs (Calvert, 1999; Schlienger et al., 1999). Therefore, training and education about ADR is particularly important.

### Research Limitations

The current research still has certain limitations. This study as a whole only covers one province in the central region, and the conclusions are extrapolated to other regions and need further research to confirm. At the same time, the study relied on pharmacists’ self-assessment of their ADR knowledge, attitudes, and practice, which may be considered a social expectations deviation, because some participants may be unwilling to reveal practice flaws. Although we used anonymity to reduce this social expectation bias during the investigation, there may be some social expectation bias because the participants may be influenced by their hospital administrators. Therefore, the true knowledge, attitude and practice of pharmacists cannot be comprehensively summarized. Despite these limitations, we believe that our research results are reliable and may help healthcare professionals improve ADR reporting in the future.

## Conclusion

Although most hospital pharmacists showed positive attitudes towards ADR reporting, their knowledge and practice were still insufficient. Hospital pharmacists’ knowledge and practice are associated with their practice towards ADR reporting. The training had a significant impact on the pharmacist’s knowledge, attitude and practice.

## Data Availability

The raw data supporting the conclusion of this article will be made available by the authors, without undue reservation.
