# Supplementary material for: Knowledge, Attitude and Practice of Hospital Pharmacists in Central China Towards Adverse Drug Reaction Reporting: A Multicenter Cross‐Sectional Study
Source: Front Pharmacol. 2022 Mar 22;13:823944. doi: 10.3389/fphar.2022.823944 (PMC8980925; doi:10.3389/fphar.2022.823944)
Supplement: Supplementary file 1 [file DataSheet1.docx]

**knowledge, attitude, and practice among hospital pharmacists towards ADR reporting**

You are receiving this questionnaire to evaluate the Knowledge, Attitude, and Practice of medication safety among hospital pharmacists . The aim of this survey is to assess the educational needs of hospital pharmacists in relevance to pharmacovigilance and adverse events reporting. Your feedback is vital to the process. We appreciate the time allocated to complete the following survey which shall take around 10 minutes of your time. Your responses will be confidential and anonymous. All responses will be compiled together and analyzed as a group.

**Characteristic**

1) Hospital type

A. Non-tertiary hospital

B. Tertiary hospital

2) Job type

A. Clinical pharmacist

B. Dispensing pharmacist

3) Gender

A. Male

B. Female

4) Age Group (years)

A. ≤35

B. 36-45

C. >45

5) Educational Qualification(s)

A. high school and below

B. college degree

C. master degree and above

6) Professional rank

A. Junior

B. Intermediate

C. Senior

7) Experience (years)

A. ≤5

B. 6-20

C. >20

8) Have you received training on adverse drug reaction reports

A. Yes

B. No

**Section A: includes 6 questions related to basic knowledge** (Correct answers)

9) Which of the following correctly defines an adverse drug reaction?

A. The harm caused by the use of inferior/counterfeit medicines

B. The harm caused by overdose

C. Harmful reactions that have nothing to do with the purpose of medication that occur under normal usage and dosage of qualified drugs

D. Adverse results related to improper drug use

E. All of the above

10) New and serious adverse drug reactions should be reported within how many days from the date of discovery or learning?

A. 1day

B. 3 day

C. 5 day

D. 15 day

E. 30 day

11) Which of the following drugs do you think will cause adverse reactions?

A. Chemicals

B. Chinese Medicine

C. Biological products

D. Diagnostic reagents

E. All of the above

12) Are all adverse drug reactions known before the drug is marketed?

A. Yes

B. No

13）Which of the following correctly defines a medical device adverse event?

A. A variety of harmful events that cause or may cause harm to the human body due to improper use of medical devices on the market.

B. A variety of harmful events that have caused or may cause harm to the human body that occur under normal use of medical devices that have been on the market.

C. Medical devices that have been on the market, due to quality problems, cause or may cause various harmful events to the human body.

D. All of the above

14) What principles should be followed for reporting adverse events of medical devices?

A. Report suspiciously

B. Report after serious damage

C. Report after confirmation

**Section B: Includes 9 questions related to hospital pharmacist's attitude.**

15) Are you concerned about the possible adverse drug reactions of patients?

A. Very unconcerned

B. Not concerned

C. generally

D. focus on

E. Very concerned

16) Do you agree that monitoring of adverse drug reactions is beneficial to public health?

A. strongly disagree

B. disagree

C. generally

D. agree

E. Very much agree

17) Do you agree that reporting an adverse reaction report can also have an impact?

A. strongly disagree

B. disagree

C. generally

D. agree

E. Very much agree

18) Do you think that reporting of adverse drug reactions is part of your responsibilities?

A. strongly disagree

B. disagree

C. generally

D. agree

E. Very much agree

19) Do you think that only serious adverse drug reactions should be reported?

A. strongly disagree

B. disagree

C. generally

D. agree

E. Very much agree

20) Do you think that the adverse drug reaction report will generate extra workload?

A. strongly disagree

B. disagree

C. generally

D. agree

E. Very much agree

21) Are you willing to participate in the training of adverse drug reaction reports?

A. Very unwilling

B. Unwilling

C. generally

D. willing

E. Very willing to

22) Do you think that monitoring of adverse drug reactions should protect patient privacy?

A. strongly disagree

B. disagree

C. generally

D. agree

E. Very much agree

23) Do you think that adverse drug reactions should be reported regularly?

A. strongly disagree

B. disagree

C. generally

D. agree

E. Very much agree

**Section C: Includes 3 questions related to hospital pharmacist's practice.**

24) In the past year, have you encountered patients with adverse drug reactions at work?

A. Yes

B. No

25）Have you reported adverse drug reactions?

A. Yes

B. No

26) What do you think is the reason that affects your report of adverse drug reactions? (multiple choice)

A. Don't know how to report

B. No time

C. Complex report

D. Think it's not part of my job

E. Lack of financial support

F. Fear of getting involved in medical disputes

G. Unable to determine whether it is an adverse drug reaction

H. Uncertainty of suspicious drugs

I. Adverse reactions are well known

J. Resistance from other stakeholders

K. Patient privacy

L. Worried about negatively affecting the unit

M. Drug batch number information is difficult to obtain
